# Tracking the evolutionary rise of C_4_ metabolism

**DOI:** 10.1093/jxb/erw137

**Published:** 2016-04-16

**Authors:** Rowan F. Sage

**Affiliations:** Department of Ecology and Evolutionary Biology, University of Toronto, 25 Willcocks Street, Toronto, ON MS3B2Canada

**Keywords:** Carbon isotope discrimination, C_3_-C_4_ intermediate species, C_4_ evolution, *Flaveria*, *F. brownii*, *F. floridana*, intermediate photosynthesis.

## Abstract

Upregulation of the C_4_ metabolic cycle is a major step in the evolution of C_4_ photosynthesis. Why this happened remains unclear, in part because of difficulties measuring the C_4_ cycle *in situ* in C_3_-C_4_ intermediate species. Now, Alonso-Cantabrana and von Caemmerer (2016) have described a new approach for quantifying C_4_ cycle activity, thereby providing the means to analyze its upregulation in an evolutionary context.

C_4_ photosynthesis is a compl**e**x trait arising from evolutionary modifications to dozens of traits in C_3_ ancestral species (Box 1). Despite this complexity, it is also one of the most convergent of evolutionary phenomena, with over 60 independent origins (Sage *et al.*, 2011). The leading hypothesis for C_4_ evolution proposes that glycine decarboxylase, a critical enzyme in photorespiration, is localized to the bundle sheath (BS) cells, thereby forcing all photorespiratory glycine to migrate from the mesophyll to BS tissues (Box 2; Rawsthorne, 1992). The resulting release of photorespired CO_2_ within the BS elevates its concentration by 200% or more, thus increasing the activity of BS Rubisco (Keerberg *et al.*, 2014). Photorespiratory glycine shuttling, or C_2_ photosynthesis as it is now termed, is thus considered to be the evolutionary bridge between C_3_ and C_4_ photosynthesis (Sage *et al.*, 2012). Because of the glycine shuttle, many features associated with C_4_ photoynthesis evolved, including Kranz-like anatomy and increased mesophyll to BS transport capacity (Sage *et al.*, 2012). In short, C_2_ photosynthesis is the foundation upon which the C_4_ metabolic cycle became established, a possibility supported by phylogenetic studies which show that C_2_ species are closely related to many C_4_ lineages (Christin *et al.*, 2011; Sage *et al.*, 2014; Fisher *et al.*, 2015).

**Box 1. C_4_ photosynthesis in *Flaveria***

The diagram shows a conceptual model of how the C_4_ photosynthetic pathway is assembled in the genus *Flaveria* (after Sage *et al.*, 2012; 2014). A successive series of traits are layered onto previously existing traits to assemble a C_4_ phenotype from a C_3_ ancestor. Key stages in the process are initial enlargement and organelle enhancement in BS cells (BSCs) to create a proto-Kranz condition. Next, restrictions of glycine decarboxylase activity to the BS creates a photorespiratory glycine shuttle that concentrates CO_2_ into the BS, enhancing Rubisco efficiency (in what is termed C_2_ photosynthesis). The C_4_ metabolic cycle is then upregulated, beginning with enhancement of PEP carboxylase (PEPCase) activity, and following a series of optimizing adaptations, an efficient, fully functioning C_4_ pathway is created. To the left of the diagram, the *Flaveria* species that correspond to specific evolutionary stages are shown, with those studied by Alonso-Cantabrana and von Caemmerer (2016) highlighted in bold. M, mesophyll.

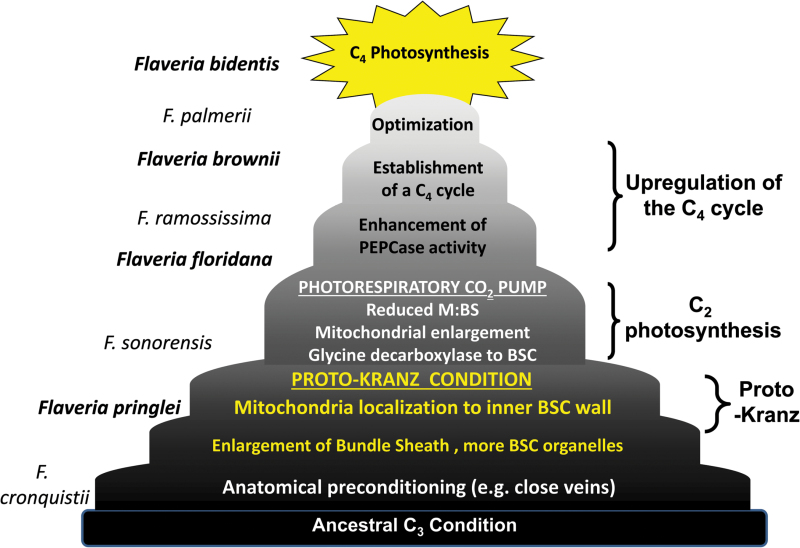

While the evidence supports a C_2_ photosynthetic bridge to C_4_ photosynthesis, it is only the first half of the bridge. The second half involves the upregulation of the C_4_ metabolic cycle. Why C_4_ metabolism became upregulated is unknown, although a recent hypothesis suggests it happened to provide carbon skeletons for re-assimilation of ammonia released by BS glycine decarboxylase (Mallmann *et al.*, 2014). A way to test this and other evolutionary hypotheses is to use a comparative approach, where the appearance of a given trait is evaluated in multiple yet distinct evolutionary lineages (Ackerly, 1999). Exploitation of the comparative approach has been facilitated by phylogenetic characterization of numerous C_4_ lineages in recent years (Sage *et al.*, 2011; Kadereit *et al.* 2012; Christin *et al.* 2013); however, given the potential numbers of lines to analyze, it is necessary to have a quick method to quantify the C_4_ cycle in an evolutionary context. This has been lacking, particularly since ^14^C-tracer methods have fallen out of favor for safety and feasibility reasons.

Using a method that involves real-time observations of steady-state ^13^C and ^12^C discrimination in plants, Alonso-Cantabrana and von Caemmerer (2016) present a rapid means to assay the contribution of C_3_ and C_4_ metabolism to carbon gain in intact leaves of C_3_, C_2_ and C_4_ species, and then test the method on four species of *Flaveria* (Asteraceae), the model genus for studying C_4_ evolution. The four species differ in degree of C_4_ cycle engagement: *F. bidentis* is a full C_4_ plant; *F. pringlei* is a C_3_ species with no C_4_ cycle activity; and two species are C_3_-C_4_ intermediates, one with a modest C_4_ cycle to compliment the dominant C_2_ cycle (*F. floridana*), the second with a strong C_4_ cycle but retaining slight Rubisco expression in the mesophyll cells (*F. brownii*, which is described as being a C_4_-like intermediate).

## Assessing C_4_ cycle activity – challenges and solutions

The challenge for assessing C_4_ cycle activity in C_3_-C_4_ intermediates is that the C_4_ cycle operates in parallel with a C_2_ cycle (which transports CO_2_ into the BS via the glycine shuttle, Box 2) and the C_3_ cycle (which is responsible for all net CO_2_ fixation in the leaf), such that the biochemical signatures of their respective activities are difficult to segregate. Historically, the relative contribution of the C_4_ cycle to carbon gain was assessed using pulse-chase experiments to determine ^14^C incorporation into the initial metabolites fixed by PEP carboxylase and Rubisco, a time consuming procedure that required sample destruction and chromatographic separation of radioactive compounds (Monson *et al.*, 1986; Moore *et al.*, 1987).

**Box 2. The photorespiratory glycine shuttle (C_2_ photosynthesis)**

The diagram summarizes the photorespiratory glycine (gly) shuttle, showing how glycolate (glc) produced after Rubisco (R) oxygenation of RuBP in the mesophyll cells diffuses into the BS cells where the glycine decarboxylase (GDC or G)-containing mitochondria can metabolize it to CO_2_, serine (ser) and ammonia (NH_3_). The CO_2_ in the BS can then accumulate to levels two to three times that in the mesophyll cells and stimulate Rubisco activity in nearby BS chloroplasts, while the ser returns to the mesophyll cells to be metabolized back to RuBP in a series of steps. A C_4_ metabolic cycle can also function in species conducting C_2_ photosynthesis, to provide additional CO_2_ to the BS, but also possibly to provide carbon skeletons to facilitate NH_3_ reassimilation in the BS, as indicated by the dashed line in the BS cell (Rawthorne, 1992; Mallmann *et al.*, 2014). glu, glutamate; HP, hydroxy pyruvate.

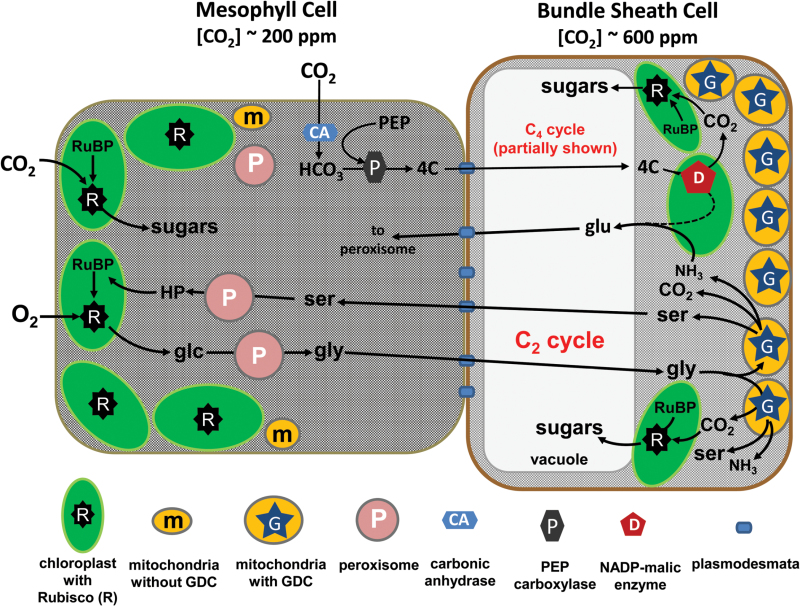

Analytical gas exchange has been widely used to identify C_3_-C_4_ intermediacy by measuring reductions in the CO_2_ compensation point of photosynthesis (Γ); however, it cannot delineate C_4_ cycle contributions because Γ is affected by glycine shuttling and the C_4_ cycle (Alonso-Cantabrana and von Caemmerer, 2016). Carbon isotope ratios (δ^13^C) can identify C_4_ cycle activity, because PEP carboxylase discriminates against ^13^C less than Rubisco. This leads to the well-known difference in δ^13^C between C_3_ and C_4_ plants, where the δ^13^C of C_3_ plants is –22‰ to –32‰ while in C_4_ plants it is –9‰ to –16‰. This difference is easily detectable with a mass spectrometer, which has been valuable for screening C_3_ to C_4_ transitions in phylogenetic clades using plant material from herbarium collections (e.g. Christin *et al.*, 2011; Fisher *et al.*, 2015).

As noted by Alonso-Cantabrana and von Caemmerer (2016), however, δ^13^C of dried plants cannot precisely delineate C_4_ metabolism in C_3_-C_4_ intermediates. Multiple processes contribute to the δ^13^C signal, including Rubisco and PEP carboxylation, refixation of photorespired CO_2_, diffusion of CO_2_ and various biosynthetic processes. Dry matter δ^13^C also integrates environmental variation during a plant’s life, and the δ^13^C in the air around the leaf can vary with position in the canopy and proximity to fossil fuel sources (an issue in urban areas, where many labs are located). To avoid these complications, real-time, mass spectroscopy should be coupled to gas exchange analyses, producing ‘on-line’ carbon isotope assessments that reflect the immediate biochemistry of CO_2_ fixation. On-line measurements are facilitated by tunable-diode laser absorbance spectrometers (TDLASs), which are best known from mesophyll conductance studies (Evans and von Caemmerer, 2013).

The on-line process factors out variation in source gas δ^13^C, producing a direct measure of discrimination (∆) against ^13^C by photosynthesis. The C_4_ versus C_3_ cycle activity can then be determined by simultaneously measuring and model-fitting CO_2_ exchange and ∆ responses to variation in atmospheric CO_2_ and O_2_, as described by Alonso-Cantabrana and von Caemmerer (2016). A key contribution of their paper is a new equation that describes ∆ responses for C_3_, C_3_-C_4_ intermediate and C_4_ photosynthesis, and incorporates contributions from mesophyll conductance and transpiration rate. This is important, because CO_2_ provided to BS Rubisco by glycine decarboxylase increases ∆, while CO_2_ provided by PEP carboxylation and the C_4_ cycle decreases ∆, such that the two signals offset. Through their approach, Alonso-Cantabrana and von Caemmerer overcome this conflict to reveal the C_4_ cycle contribution.

## New measurements with *Flaveria*

With their approach, Alonso and von Caemmerer (2016) estimate that C_4_ cycle activity at current atmospheric CO_2_ levels contributes about 12% of the carbon assimilated in *F. floridana* and 80% of the carbon assimilated by *F. brownii* (see Fig. 8 in Alonso-Cantabrana and von Caemmerer), which is comparable to pulse-chase estimates using ^14^C (Moore *et al.*, 1987). Of note, they are able to examine the change in the C_3_ and C_4_ contributions across a range of intercellular CO_2_ levels in the same leaf. Thus, for example, at CO_2_ levels approximating pre-industrial values (280ppm), the C_4_ cycle contribution increases to 15% in *F. floridana* and 90% in *F. brownii*. At high CO_2_, the C_4_ contribution dropped off in *F. brownii*, to only 75%, reflecting a marked increase in the efficiency of the residual Rubisco left in its mesophyll tissue. This increase in the contribution of Rubisco to CO_2_ fixation causes a substantial rise in the biochemical ∆ from below one at low CO_2_ to near six at high CO_2_ (Fig. 5 in Alonso-Cantabrana and von Caemmerer). Moreover, the ability to predict the CO_2_ response of photosynthesis in the intermediates was much improved by incorporating their estimated C_4_ cycle contribution, as was the modelled CO_2_ response of ∆.

These responses highlight how small amounts of C_4_ cycle activity can improve carbon gain at low CO_2_ levels, yet become less significant at elevated CO_2_ if a modest amount of Rubisco is present in the mesophyll tissue. In *F. floridana*, the function of the C_4_ cycle has been questioned, since it seemed to contribute little to photosynthesis, and thus was suggested to initiate a futile cycle (Monson *et al.*, 1986). Alonso-Cantabrana and von Caemmerer demonstrate that the C_4_ cycle does indeed contribute to CO_2_ fixation, and thus is not futile and could be adaptive in low CO_2_ conditions of recent geological time, when atmospheric CO_2_ fell below 200ppm (Gerhart and Ward, 2010).

In summary, Alonso-Cantabrana and von Caemmerer have provided researchers with a powerful approach that can be quickly applied to many C_3_-C_4_ intermediates from a range of lineages, thereby enabling comparative analyses for addressing hypotheses explaining how evolution upregulated C_4_ metabolism. When coupled with genomic and ecological data, C_4_ researchers should now be able to evaluate in detail how one of the most evolutionary complex traits on Earth repeatedly evolved in recent geological time.
